# Comparing Business Experts and Novices in Complex Problem Solving

**DOI:** 10.3390/jintelligence5020020

**Published:** 2017-05-13

**Authors:** C. Dominik Güss, Hannah Devore Edelstein, Ali Badibanga, Sandy Bartow

**Affiliations:** 1 Department of Psychology, University of North Florida, Jacksonville, FL 32224, USA; hannah.devore@gmail.com (H.D.E.); ali.badibanga@unf.edu (A.B.); 2 Jacksonville Chamber Foundation, Jacksonville, FL 32202, USA; Bartowsandy@gmail.com

**Keywords:** expert-novice differences, crystallized intelligence, flexibility, adaptability, complex problem solving, dynamic decision making, virtual environment

## Abstract

Business owners are faced with complex problems and are required to make decisions on a daily basis. The purpose of this study was to investigate complex problem solving (CPS) between experts and novices and to explore the competing theories of expert-rigidity versus expert-adaptability, as part of exploring which theory better explains crystallized intelligence. Participants were 140 business owners, business management undergraduate students and psychology students. Each participant managed a highly complex simulated chocolate company. Decisions and systems data were automatically saved in log files. Results revealed that small business owners performed best, followed by business students and then psychology students. A process analysis revealed that experts compared to novices spent more time initially exploring the complex situation. Experts were found to have greater flexibility in their decisions, having made the most personnel and advertising changes in response to situational demands. Adaptability and flexibility were predictive of performance, with results supporting the adaptability/flexibility theory of expertise. This study shows the influence of expertise on complex problem solving and the importance of flexibility when solving dynamic business problems. Complex business simulations are not only useful tools for research, but could also be used as tools in training programs teaching decision making and problem solving strategies.

## 1. Introduction

How does an experienced business leader compared to a novice balance production numbers and demand on the market? Which strategies do experts and novices use to expand their company’s position in the market? Are business experts reacting to changes in the market with flexibility? Do experts and novices differ in their information collection strategy? The current study investigates complex problem solving (CPS) behavior in a complex and uncertain, business simulation of a chocolate company called CHOCO-FINE. The research focus was to empirically investigate the role of expertise in CPS strategies by comparing experts and novices over time, contributing to the literature on prior knowledge in CPS as outlined in the editorial to this Special Issue.

### 1.1. Complex Problem Solving and Dynamic Decision Making under Uncertainty Using Micro-Worlds

The first attempts to use simulations pertaining to complex and uncertain problems as part of investigating participants’ decision making occurred some years ago. In Europe, computer-simulated complex problems, also called micro-worlds or virtual environments, were developed to investigate how participants dealt with uncertainty and complexity [1,2,3,4,5]. This research technique became popular under the label complex problem solving (CPS) [6].

In the United States, similar research using simulated problems also increased in popularity, developing under the label dynamic decision making (DDM) [7,8]. Since then, these micro-world simulations have been frequently used for research in the field of business [9,10].

In both the CPS and DDM traditions [11], simulated problems include certain characteristics [12]. First, simulated problems are dynamic, i.e., they change over time, and new decisions are dependent on previous decisions. Second, they are complex and consist of many interconnected variables. The complexity of these variables is dynamic and unpredictable, increasing challenges for predicting future developments in the micro-world. Third, the simulated micro-worlds are non-transparent, meaning that neither the structure of the variables, nor the dynamics are fully disclosed to the participants. Dynamics, complexity and connectivity, as well as non-transparency ultimately lead to the decision maker feeling uncertain.

Frequently, management strategies are investigated using case studies, retrospective analyses of historic data, the scenario technique and self-reports to evaluate decision outcomes; among these, survey type measures are the most widely used [13]. One limitation of these methods is their focus on the one moment and the neglect of time dynamics and complexity. To be successful in micro-worlds, for example, one must learn to adapt and remain open to various decisions [14]. The study of expert problem solving and decision making in the industrial/organizational context, with a focus on a process analysis in realistic contexts, is highly relevant and has been advocated by many researchers [15]. The use of such micro-worlds is a technique that allows researchers to investigate CPS over time, increasing the external validity of the findings when compared to existing research on CPS that applies self-report techniques and focuses on static problems.

### 1.2. Expertise and Crystallized Intelligence

As previously mentioned, the focus of the current study is to empirically investigate the role of expertise in CPS over time. The topic of expertise is related to this special issue on intelligence and CPS, because expertise is highly related to crystallized intelligence. Crystallized intelligence refers to knowledge and experience learned in a specific cultural environment [16,17]. In a knowledge-rich problem situation such as CHOCO-FINE, the problem solvers rely on and apply knowledge they have acquired in the past and stored in long-term memory; thus, CPS depends heavily on crystallized intelligence. A meta-analysis [18] revealed that crystallized intelligence is a strong predictor for job performance. In the current study, we operationalized expertise as experience and knowledge regarding business management, comparing experts, semi-experts and novices. Developing expertise can be defined as the “ongoing process of the acquisition and consolidation of a set of skills needed for a high level of mastery in one or more domains of life performance” ([19], p. 359). In this sense the term expertise is highly related to crystallized intelligence.

### 1.3. Expertise and CPS Performance

In many areas, such as business management, expert level performance cannot be accounted for by stable heritable characteristics, but instead can be attained through learning and deliberate practice [20]. Ericson et al. [20] define deliberate practice as “a regimen of effortful activities” (p. 363), as engaging in activities in a specific domain over many years with the goal of improving performance. The expert to be has to show perseverance and motivation to excel and needs to receive feedback about his or her performance allowing for room to learn and improve.

It is self-evident that experts perform better in their domain of expertise than novices, and there are several reasons why experts are more successful. First, experts have more elaborate knowledge in their domain of expertise; both declarative and procedural knowledge. Second, experts know, due to their procedural knowledge, what decisions to make, when to make decisions and when to change their decision-making approach. As part of their declarative and procedural knowledge, experts perform better at identifying possible solutions compared to novices [21]. Third, experts know what aspects of the problem have to be explored and what cues to focus on. These three factors are highly interwoven when solving complex problems, particularly when decisions directly affect and change the problem situation [22].

### 1.4. Rigidity versus Flexibility in CPS

It is unclear, however, if expertise leads to rigidity or flexibility in CPS. Does crystallized intelligence or expertise make someone’s decisions more rigid or more flexible? Research on expert-novice comparisons has shown contradictory findings. Some theories, such as the rigidity model, suggest that an increase in expertise is related to an increase in rigidity [14,23]. Other theories, such as the deliberate practice theory, suggest that with an increase in expertise, there is an increase in adaptability and flexibility [24,25].

Two additional components of knowledge requiring discussion are declarative and procedural knowledge. One aspect about expertise is that it is characterized by enormous declarative knowledge. Declarative knowledge refers to facts and informational knowledge about a specific domain. This type of knowledge is stored and organized in elaborate schemata [26]; or templates [27]; or action schemata as described in PSI-theory [28] (the name PSI comes from the Greek letter *Psi* which is often used as an abbreviation for Psychology). Previous research on experts has found that they have larger and more complex schemata pertaining to a specific domain [14,29].

Another aspect related to expertise is procedural knowledge. Procedural knowledge is the knowledge of how to perform tasks and activities. This knowledge often requires problem solving strategies, specific to a task (see, e.g., for the field of sport, [30,31]). When applying procedural knowledge, an individual may consider: What shall I do first? What information is crucial? What are the consequences, if I do this versus that? 

Both experts’ depth of declarative and depth of procedural knowledge influence their problem solving. It is unclear, however, if declarative and procedural knowledge might lead to more “fixed” paths or allow the development of multiple perspectives and flexibility in approaching a task; an increase in perspectives may lead to a more flexible problem solution and decision making.

What evidence speaks for rigidity in experts’ decision making? When experts emphasize their reliance on past experience and developed schemata to make decisions, they often apply “old” decisions to the current problem, failing to acknowledge essential differences between old and new situations. The literature refers to this inflexible decision making behavior as a mental set failure [32,33], the Einstellung effect [34,35] or methodism [12].

What evidence speaks for flexibility in experts’ decision making? Experts’ elaboration of declarative and procedural knowledge consists not only of one-way solutions, but often of a number of possible alternative solutions (see, e.g., [22,36,37]). This allows flexibility in reasoning, which is especially important in “ill-structured” domains [36,38], such as management. In experiments, where, for example, experts and novices were compared in the two different domains of management and English grammar, experts were less rigid and showed more mental flexibility in their problem solving and decision making than novices [25].

In sum, there is some evidence speaking for the rigidity of experts and some evidence speaking for the flexibility of experts. In the current study, the flexibility and rigidity of experts and novices will be studied in the dynamic process of decision making and CPS.

### 1.5. Expertise and Problem Exploration

Gathering information and exploring the problem is a crucial step in CPS and allows for the development of a mental model of the situation [12]. In some domains, such as fire-fighting, it is absolutely necessary to make fast decisions, as well as to collect information about an emergency, such as a large wildfire emergency (we will come back to this point later). In domains such as marketing, politics and engineering, it is more prudent to spend time exploring the situation and to identify resources and strategies before making decisions [39]. When confronted with complex problems, research indicates that experts engage in greater in-depth analysis of the situation than novices [40]. As the situation further develops, people have already developed a mental model of the situation and require less information for their strategic decision making.

Experts have been shown to search in broader and greater depth for information [41] and to fall less often for the confirmation bias than novices in situations with conflicting information [42]. Due to experts’ elaborate schemata, they know which aspects of the problem situation are most relevant and can discern between unnecessary and necessary information when problem solving [43]. In contrast to novices, experts explore a broader problem space [44].

### 1.6. Research Questions

The goal of the current study is to extend previous research and to focus on actual CPS behavior through the use of the complex and uncertain micro-world CHOCO-FINE. The study will focus on crystallized intelligence and successful and failing strategies of novices and experts, further exploring the contradicting theories on expert rigidity versus flexibility. Based on the research discussed, the following four research questions were developed:

1. How will performance differ between experts, semi-experts and novices in the CHOCO-FINE micro-world simulation?
2. Are there differences in rigid and flexible decision making between experts, semi-experts and novices?
3. What are the differences between experts, semi-experts and novices in their initial search and exploration for information in the CHOCO-FINE micro-world simulation?
4. What are the effects of in-depth exploration and flexibility on CPS performance?

## 2. Materials and Method

### 2.1. Participants

Participants were 72 psychology undergraduate students (novices), 50 business undergraduate students from the same university in the Southeastern United States (semi-experts) and 28 small business owners (experts) from the community where the university is located. Data of three participants were not included (2 psychology students and one business owner) because they had in Month 1 already −$3,000,000,000 total monies, probably due to input mistakes. Data for seven participants were not saved or stored automatically due to computer problems resulting in a total of 140 participants: 66 psychology undergraduate students (novices), 49 business undergraduate students (semi-experts) and 25 small business owners (experts). Participants’ ages ranged from 18 to 59 years (*M* = 28.69, *SD* = 11.95) and varied between groups, *F*(2, 137) = 90.25, *p* < .001, *ƞ*^2^_p_ = .57 (business owners *M* = 47.92, *SD* = 8.10; business students *M* = 25.06, *SD* = 8.08; psychology students *M* = 24.09, *SD* = 7.71).

Regarding gender, 63% of all participants were female and 37% were male. The gender distribution also differed significantly among the three groups, χ^2^ (2, *N* = 140) = 29.81, *p* < .001. Gender distributions for the respective groups were as follows: small business owners (84% female and 16% male), business students (33% female and 67% male) and psychology students (77% female and 23% male). Since performance in CHOCO-FINE, i.e., total monies at Month 19, neither correlated significantly with gender (*r* = .10, *p* = .26), nor with age (*r* = .13, *p* = .14), gender and age were not included as covariates in the following analyses. We only include age and gender in the regression analyses to test Research Question 4.

All subjects gave their informed consent for inclusion before they participated in the study. The study was approved by the Ethics Committee of the University of North Florida Institutional Review Board (IRB# 10-059).

### 2.2. CHOCO-FINE Simulation

The micro-world used for the current research should be knowledge-rich and reflect the complexity of the business world, to some degree, including areas such as market, competitors, advertising, staffing, production and product profiles. We chose the highly complex, uncertain and dynamic simulation CHOCO-FINE for the current study. It was originally developed by Dörner [45] in cooperation with experts from the business field. The version used for the proposed study is the third revised version of the simulation and contains more than 1000 simulated variables in 19 domains (e.g., personnel, marketing, production). Each participant takes the role of the CEO of a chocolate-producing company called CHOCO-FINE and participates in the role over a period of time.

**Figure 1:**
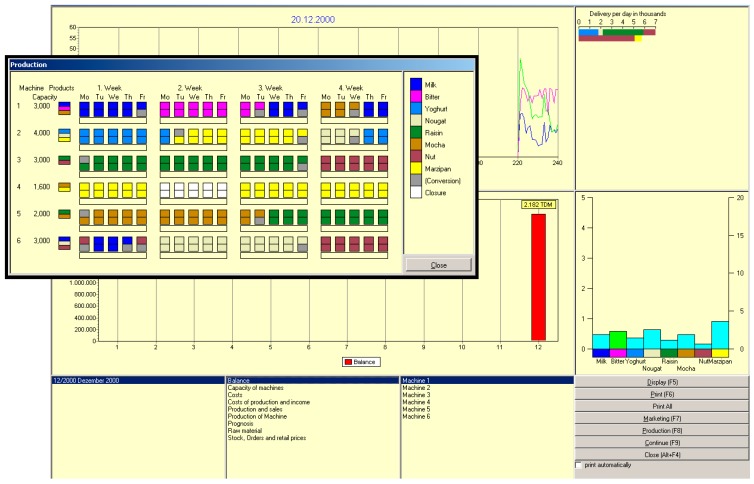
Production screen in front of the main screen of CHOCO-FINE.

For each simulated month, the participant gathers information and makes decisions effecting the success of their company. The participant can analyze changes that occurred, gather further information, make decisions and proceed to the next month. The user interface of the program consists of 3 screens (see Figure 1): (1) the main screen; (2) the production screen (which is shown here in reduced size in front of the main screen); and (3) the marketing screen (not shown). The main screen shows basic data and information, such as production, demand, sales, account balance, deliveries per day, stock of inventory and open orders.

The production screen shows 6 machines, their capacities per half day, which kinds of chocolates can be produced on each of them and the 20 work days of the month, divided into 2 half days. The marketing screen shows a stylized city map with the different districts and quarters shown in pie charts. The size of the pies corresponds with the sizes of the local market. The marketing window provides several options to gather information, conduct market research and make decisions regarding advertising, product design, prices or the hiring of sales representatives.

After an initial 30 min to become familiar with the simulation, each participant worked individually on the CHOCO-FINE simulation for almost 2 h. An instruction sheet describing the CHOCO-FINE simulation was distributed to each participant and kept for the duration of the simulation. The variables were operationalized in the following way.

(1) Performance: Performance was operationalized as total monies at the end of each month. Total monies at Month 19 was chosen as the performance variable for the correlations.
(2a) Decision-making/problem-solving areas: The focus on specific decision-making areas was operationalized through the amount of money spent during each month for each of the three decision-making areas: advertising, personnel and market research. For advertising, participants could have chosen general overall brand advertising or specific advertising for specific chocolates or specific product profile components. For market research, participants had access to view and purchase information related to their own products and clients, as well as their competitor products and clients. Regarding personnel, participants had expenses, such as salaries, hiring, firing and relocating.
(2b) Decision-making changes: The changes in each specific decision-making area were operationalized through the number of changes from one month to the next month relative to the total number of months completed. Change were coded as binary, either as a “0” if no change happened from one month to the next month, or as “1” if a change happened. For example, 10 changes in representatives in 19 months would result in .53.
(3) Exploration: Depth of exploration was operationalized as time spent working on the first two months of the simulation. Participants were able to control proceeding to the next month of the simulation by clicking the “continue” button after all decisions for the month were made. At first, we considered only taking the first month, but since 20% of all participants spent less than 10 min for both months and since the first month also requires adjustment to the screens, we decided that taking the first two month would provide a clearer picture on exploration.

### 2.3. Demographic Survey

After completion of CHOCO-FINE, participants received a demographic survey including questions referring to gender, age, education and professional background.

### 2.4. Procedure and Data Analysis

The participants were given a brief introduction to the three computer screens and commands of the simulation, followed by 20 min to explore the simulation with an instructional guide. Once the simulation was restarted, participants began working in their role as CEO, participating in the simulation for an average of two hours.

Every single decision a participant made during CHOCO-FINE was automatically saved in computer files. The CPS strategies were operationalized (see above) and tallied from the participants’ saved computer files by the second and third author (a similar procedure was used by Güss, Tuason, and Orduña [46], although focusing on different strategies and errors in the different simulation WINFIRE). After completion of the CHOCO-FINE simulation, participants were given a short demographic survey. Business owners received additional questions regarding the simulation’s validity including questions regarding the realism of CHOCO-FINE and its specific parts: market, products, production, competitors, clients, pricing, advertisement, research and development. Ratings on 5-point Likert scales indicated either high or low realism for the different parts of the micro-world. A sample question was, “Do you think CHOCO-FINE reflects realistic aspects of the market?”

## 3. Results

### 3.1. Face Validity of CHOCO-FINE

One indicator of the face validity of CHOCO-FINE is the results from the survey distributed to the business owners. As mentioned previously, the survey refers to the simulation’s overall realism through its eight parts: market, products, production, competitors, clients, pricing, advertisement, research and development. Results of the ratings of the eight parts on the five-point Likert scales indicate medium to high realism of the eight different micro-world parts (*M*s between 3.1 and 3.8).

### 3.2. Expertise and Performance

Research Question 1 investigated the performance differences between experts, semi-experts and novices in the CHOCO-FINE micro-world simulation. A mixed between-within subjects analysis of variance (ANOVA) was run to investigate differences among the 19 months and among the three groups (business owners, business students and psychology students) in total monies. From the 140 participants, total monies of one month for one person were removed because they were less than −$9 million. The interaction between months and groups was significant, Wilks’ Lambda = .59, *F*(36, 216) = 1.82, *p* = .005, *ƞ*^2^_p_ = .23. The main effects are therefore qualified. The main effect of months was significant, Wilks’ Lambda = .29, *F*(18, 108) = 14.67, *p* < .001, *ƞ*^2^_p_ = .71, indicating that overall, participants lost money; over time, performance decreased. The main effect for expertise was significant, *F*(2, 125) = 3.10, *p* = .049, *ƞ*^2^_p_ = .05, indicating that business owners lost less money compared to business and psychology students.

We also analyzed the performance data using growth mixture modeling in the Mplus software [47] to compare the trajectories of the three groups. This procedure allows a comparison of the slopes and intercepts of the performance trajectories of the three groups (see [48] for a detailed description of the methodology). None of the intercepts and slopes differed significantly among the three groups: *z*-score for business owners and business students comparison of intercept = 0.06, of slope = 0.30; *z*-score for business owners and psychology students comparison of intercept = −0.17, of slope = 0.63; *z*-score for business students and psychology students comparison of intercept = −1.09, of slope = 1.84.

**Figure 2:**
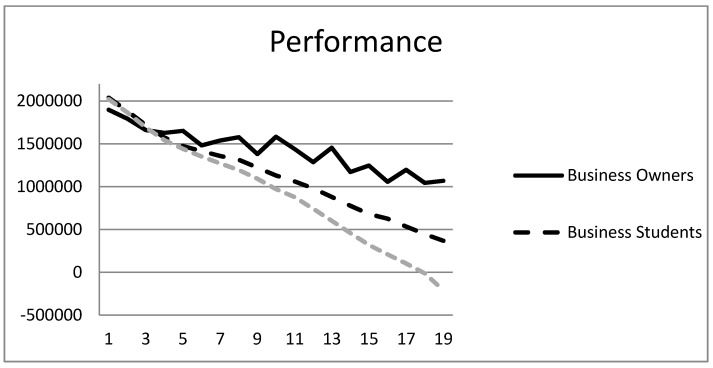
Account balance means for each of the 19 months of CHOCO-FINE for the three samples.

As Figure 2 shows, and as expected based on research on experts and novices, business owners performed best, followed by business undergraduates and psychology undergraduates, in that order.

The results also showed how difficult it is to manage the complexity of CHOCO-FINE for all participants. The account balance range of individual participants was from −5.8 million to +3.6 million. Only 9% of all participants had an account balance higher than 2.1 million in Month 19, thus improving the company’s balance compared to the initial situation of 2.1 million.

### 3.3. Rigidity versus Flexibility in CPS

Research Question 2 investigated if there are differences in rigid and flexible decision making between experts, semi-experts and novices, further exploring the expertise-rigidity model and the expertise-flexibility model and investigating: (a) the focus on different areas in CHOCO-FINE; and (b) the changes in decision making.

(a) Three main areas were investigated; advertising, market research and personnel.

**Figure 3:**
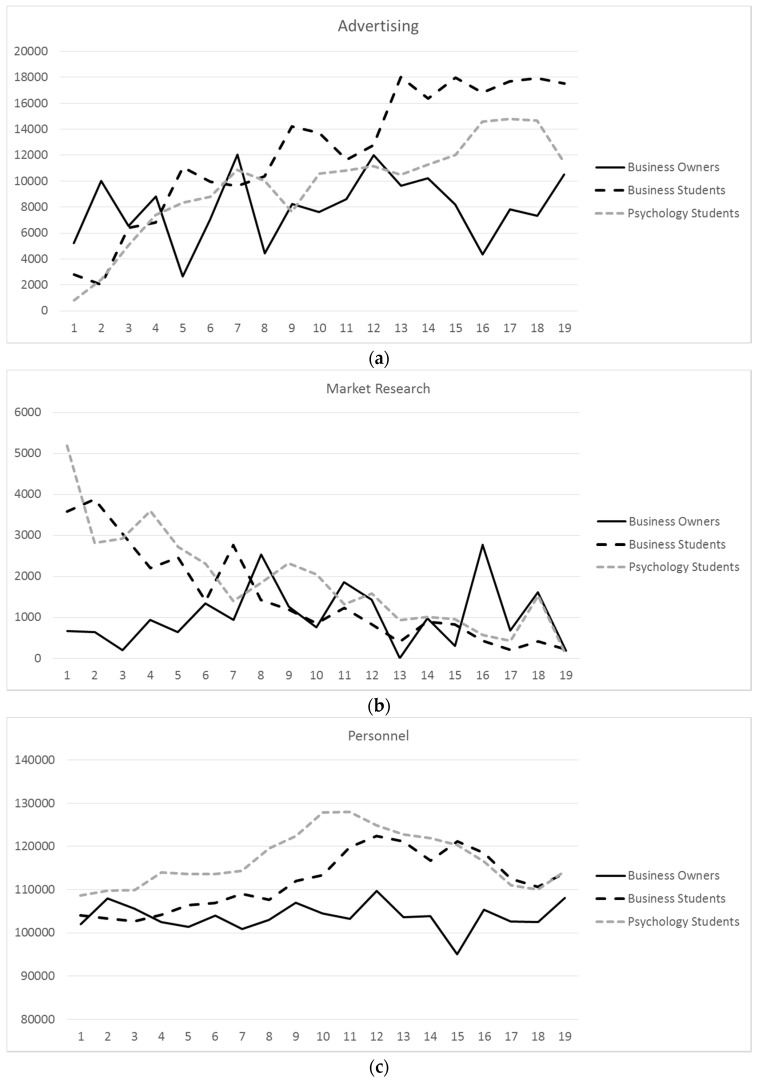
(**a**) Advertising, (**b**) market research and (**c**) personnel expenses’ means for the 19 months of CHOCO-FINE for the three samples.

A mixed between-within subjects ANOVA was run to investigate differences among the 19 months and among the three groups in advertising expenses (see Table 1 and Figure 3). Thirty-six extreme outliers out of 2660 (19 months times 140 participants) were identified, resulting in excluding advertising months with monthly expenses greater than $180,000. The interaction between months and groups was significant. The main effect of months was significant; advertising expenses increased over time. There was no main effect for expertise, as the three groups did not differ in advertising expenses.

When comparing only the main effect for group in advertising expenses in the first three months, *F*(2, 136) = 3.61, *p* = .03, *ƞ*^2^_p_ = .05, the three groups differed significantly. Pairwise comparisons showed that business owners spent significantly more than business students (*p* = .02) and psychology students (*p* = .01). Business students and psychology students did not differ from each other (*p* = .84).

To compare expenses for market research, a mixed between-within subjects ANOVA was run to investigate differences among the 19 months and among the three groups (see Table 1 and Figure 3). Thirteen extreme outliers out of 2660 were identified, resulting in excluding market research months with expenses greater than $33,000. The interaction between months and groups was not significant. The main effect of months was significant. Over time, expenses decreased. The main effect for expertise was not significant.

When comparing only the main effect for group in market research expenses in the first three months, *F*(2, 132) = 7.01, *p* = .001, *ƞ*^2^_p_ = .10, the three groups differed significantly. Pairwise comparisons showed that business owners spent significantly less than business students (*p* = .001) and psychology students (*p* = .001). Business students and psychology students did not differ from each other (*p* = .90).

Comparing personnel expenses, a mixed between-within subjects ANOVA was run to investigate differences among the 19 months and among the three groups (see Table 1 and Figure 3). Twenty-five extreme outliers out of 2660 were identified, resulting in excluding personnel expenses months with expenses greater than $500,000 or below $500. The interaction between months and groups was not significant. The main effect of months was significant. The main effect for expertise was not significant.

When comparing only the main effect for group in personnel expenses in the first three months, *F*(2, 136) = 1.79, *p* = .17, *ƞ*^2^_p_ = .03, the three groups did not differ significantly.

We additionally conducted growth mixture modeling in the Mplus software [47] to compare the trajectories of the three groups in advertising, market research and personnel expenses (see [48] for a detailed description of the methodology). None of the intercepts and slopes in the three areas differed significantly among the three groups with one exception. Psychology students spent significantly more than business students for personnel as shown in the significant *z*-score comparing the intercepts for personnel expenses, *z* = 10.546, *p* < .001.

In sum, whereas spending varied among the 19 months, experts did not differ significantly from novices in expenses for advertising, for market research and for personnel. Differences only appeared when comparing expenses for the first three months; business owners spent less for market research, but more for advertising compared to business students and psychology students. When comparing the means of all expenses for Months 1 to 19, the three groups did not differ in advertising, *F*(2, 137) = .59, *p* = .56, *ƞ*^2^_p_ = .01; did not differ in personnel, *F*(2, 136) = 1.47, *p* = .23, *ƞ*^2^_p_ = .02; but did differ marginally in expenses for market research, *F*(2, 137) = 2.47, *p* = .09, *ƞ*^2^_p_ = .04.

(b) Changes in decision making.

**Figure 4:**
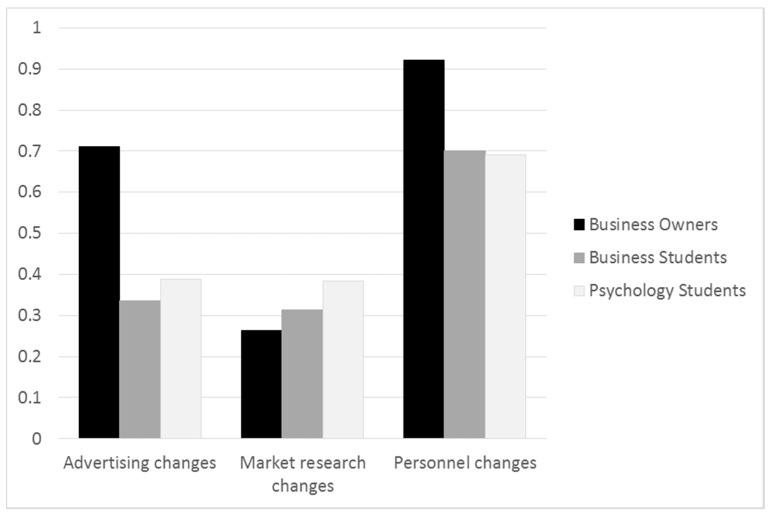
Number of changes relative to number of CHOCO-FINE months played for the three areas, advertising, market research and personnel, among business owners, business students and psychology students.

Experts compared to novices are expected to change their CPS approach more often and adapt to changing situations. We calculated the number of changes in the three areas of advertising, market research and personnel (indicated by changes in their monies spent for each area in each month) and divided it by the number of months completed. A binary value of one indicated that in each of the months, the participants made changes (see Figure 4).

Regarding advertising, a one-way between-groups ANOVA showed that the three groups differed significantly in the proportion of months they made advertising changes. Pairwise comparisons with Bonferroni adjustment showed that business owners made more changes compared to both psychology students (*p* < .001) and business students (*p* < .001). Psychology and business students did not differ significantly from each other (*p* = .78). Regarding market research, a one-way between-groups ANOVA showed that the three groups differed significantly in the proportion of months they made market research changes. Pairwise comparisons with Bonferroni adjustment showed that business owners made significantly fewer changes than psychology students (*p* = .03). Psychology and business students did not differ significantly from each other (*p* = .19), as well as business students and business owners (*p* = .89). Most participants, however, did not search for and spend money towards market research. Regarding personnel, a one-way between-groups ANOVA showed that the three groups differed significantly in the proportion of months they made personnel changes. Pairwise comparisons with Bonferroni adjustment showed that business owners made more changes compared to both psychology students (*p* < .001) and business students (*p* < .001). Psychology and business students did not differ significantly from each other (*p* = 1.00).

### 3.4. Initial Problem Exploration

Information in CHOCO-FINE (such as account balance, capacity of machines, costs for production, production numbers, sales numbers or raw material) is free and provided on the main screen. For specific information regarding the market, products, competitors and customers, participants have to access the market research screen and pay for the information. Research Question 3 explored if there are differences between experts, semi-experts and novices in their initial search and exploration for information, therefore spending more time in the first two months of CHOCO-FINE to explore the simulation. A one-way between-groups ANOVA showed that exploration time differed significantly between groups. Pairwise comparisons with Bonferroni adjustment revealed that business owners (*M* = 27.60, *SD* = 12.51) spent significantly more time than business students (*M* = 20.36, *SD* = 13.03), *p* = .03, and spent significantly more time compared to psychology students (*M* = 18.01, *SD* = 9.74), *p* = .002, but business students did not differ from psychology students (*p* = .84).

### 3.5. Predicators of CPS Performance

Research Question 4 investigated the performance effects of in-depth exploration and flexibility in the decision making CPS strategy. To answer this, we calculated correlations and partial correlations (controlling for age and gender) between performance and CPS strategies (see Table 2). Mean expenses in advertising and personnel correlated negatively with performance, i.e., total monies. Changes in advertising and personnel correlated positively with performance.

A multiple regression was run to detect the influence of the variables on performance. Tolerance values were between .44 and .93, and variance inflation factors VIF were between 1.08 and 2.27, revealing that multicollinearity was not a problem. The model including the seven variables was significant, *F*(10, 116) = 4.37, *p* < .001, explaining 27% of the variance in performance (*R*^2^ = .27). Gender, expenses for personnel and personnel changes significantly predicted performance (see Table 3). Comparing overall performance between male (*N* = 51, *M* = 366,596.86, *SD* = 1,597,569.14) and female participants (*N* = 78, *M* = 20,664.79, *SD* = 1,759,790.69), however, did not show significant differences, *t*(127) = −1.13, *p* = .26.

## 4. Discussion

The goal of the current study was to extend previous research and to focus on the following: (a) actual CPS behavior in a complex, uncertain and dynamic business micro-world; (b) to investigate those CPS strategies in business experts and novices empirically and over time, addressing the potential influence of crystallized intelligence on CPS; (c) to explore the competing theories on expert-rigidity versus expert-flexibility; and (d) to explore the role of problem exploration in CPS.

Regarding Research Question 1, experts performed better than novices in CHOCO-FINE. As expected, business owners performed best, followed by business students and followed by psychology students. It is noteworthy, though, that 90 percent of all participants had problems managing the chocolate company and lost money. One reason could be the lack of market research considered by participants. In most months, two thirds of the participants did not inquire market research information regarding their own product profiles, their own clients, their competitors and their competitor’s products. The importance of information searching has also been shown to improve performance in other areas, such as with tax professionals [49]. We will come back to the lack of market research later. The better performance of experts compared to semi-experts and novices coupled with the medium to high realism ratings of business owners in the survey are indicators for the face validity of the CHOCO-FINE simulation.

Research Question 2 further investigated the competing theories of expert rigidity versus expert flexibility. First, we compared the expenses in advertising, market research and personnel among the three groups over 19 months. The three groups never differed. Comparing only the first three months, business owners spent more money on advertising and less on market research compared to business and psychology students. They recognized how crucial advertising is. Yet, they later spent less money on advertising than business students and psychology students. Potentially, their advertising has become more targeted and focused on specific products or clients. Regarding market research, business owners spent less than business students and psychology students. One reason could be that they relied on their experiences and knowledge regarding the market. The differences in their strategic approach might again be due to differences in declarative and procedural knowledge between novices and experts. One could interpret the finding of differences in the first three months and none over the entire 19 months as differences in cognitive schemata between business owners, business students and psychology students. Schematic differences may have influenced the CPS approach more so earlier in the simulation than later on. In the second half of the simulation, the situation characteristics of CHOCO-FINE may have been more dominant and “overpowered” the influence of the schemata on decision making.

Concerning changes in their strategies from month to month, there were significant differences in advertising changes, market-research changes and personnel changes among the three groups. Regarding market research, business owners made fewer changes than psychology students. Yet, since almost two thirds of all participants did not spend money for market research in a given month, market research in CHOCO-FINE did not play a central role among most participants. Whereas business owners changed their approach to advertising and personnel almost every month and adapted their decisions to the current situation, business and psychology students tried to follow a multi-month strategy and changed their decision-making approach every other or every third month. Decision making that follows the same approach and is not changed when the situation changes has been called methodism [12] and can lead to failure. These findings speak to the adaptability/flexibility models of expertise [25].

Lack of flexibility is typically related to an increase in expertise and more rigid schemata as some researchers claim [14]. In the current study, business owners did not show this lack of flexibility or fall for methodism. To the contrary, they mostly demonstrated flexibility. A business owners’ sensitivity and adaptability to changes in the environment has also been discussed in other research on management as key to success [50]. One possible explanation for the sensitivity and flexibility of business owners could be self-monitoring and self-reflection. Experts have better information processing speed and excellent self-monitoring ability [51], and research has shown that managers with greater metacognitive skills make less erratic strategic decisions [52]. Business owners might be more aware and sensitive to surprising or contradicting new information. These can lead to increased self-reflection, such as, “What has happened?”, “What caused this situation?” and “What do I have to do now?” Furthermore, interviews with small business owners revealed that among the key skills mentioned in performance improvement were stress management, practicing self-control and engaging in behavioral changes [53].

Research Question 3 investigated whether experts spent more time than novices gathering initial information during the first two months of CHOCO-FINE as part of exploring the simulation. A detailed problem analysis is necessary when dealing with a complex, dynamic and uncertain problem situation, such as CHOCO-FINE [54]. Indeed, business owners spent more time than business students and psychology students in their initial exploration. This is another finding that suggests that business owners as experts did not fall for methodism or a given mental set, but were willing to explore the new situation in much more detail before acting when compared to business students and psychology students. Information has to be integrated in a meaningful way into mental models for the situation and ultimately lead to promising decisions [55,56]. As performance in CHOCO-FINE shows, business owners were successful at finding the key information and integrating it with their mental models; or they could not adjust decisions adequately to the provided information.

The differences in exploration time refer more to the discovery of the company’s general information (such as production, demand, sales, account balance, deliveries, stock of inventory and open orders) and not to specific market research (clients and competitors), because the three groups did not differ significantly in their market research expenses for the first three months. Perhaps it is not too surprising that business and psychology students did not conduct much market research, but why did the business owners not engage in more market research? One reason could be related to sample characteristics of the business owners in the current study. All business owner participants were small business owners. It is possible that in their businesses, market research is not as crucial compared to mid-size or larger companies. Small business owners have direct contact with their customers and therefore receive direct feedback from them. Future research could compare small business owners with managers/executives from large companies to clarify the relationship between market research and the size and kind of business.

The more in-depth search for information of experts was also shown in other research. Strohschneider and Schaub [40], for example, found in their comparisons of managers and students in dynamic micro-worlds that managers explored the simulations in the first months longer than the students and could resist, for longer periods of time, the impulse to act in comparison to the students. It could be that psychology students in our study did not realize the importance of business-related information and could not integrate it in existing schemata. It could also be that psychology students were overwhelmed by CHOCO-FINE and could not tolerate the uncertainty and therefore felt a stronger need to prove themselves and to act, i.e., to show themselves that they can deal competently with this situation, which can lead to “thematic vagabonding” [12], i.e., to jump from one area to another without following a detailed plan.

Although the regression analyses did not provide strong evidence, and we have to acknowledge the low power due to the relatively small sample size, it is exactly the changes in CPS that are predictive of overall performance (Research Question 4). In a complex and dynamic situation like CHOCO-FINE, one must observe the situation in detail (see also, e.g., [57]; one must look at the changes in sales and adjust production numbers; and one must be prepared to react to new products of competitors by changing the advertisement. This flexibility and adaptability to situational changes is exactly the opposite of methodism and rigidity, i.e., the tendency to always do what one has done previously.

### Limitations and Future Directions

One potential limitation of the current study is the focus on the decision logs of CHOCO-FINE. Future research could ask participants to think-aloud while working on CHOCO-FINE. These thinking-aloud protocols or verbal protocols could reveal if experts engage, indeed, in more self-reflection compared to novices [58,59]. They could also reveal which events or information in CHOCO-FINE triggered their changes in CPS.

Another limitation of the current study are demographic differences among the three samples. Although our samples obviously differed in relation to business knowledge and most likely crystallized intelligence, we did not control for prior subject matter knowledge and have not assessed crystallized intelligence [60,61].

The benefit of simulations to study learning has been expressed repeatedly, e.g., “Traditionally, knowledge is assessed by verbal achievement tests on the subject matter. However, traditional methods are regarded as limited in their ability to assess higher-order learning or understanding” (Burkolter, Meyer, Kluge, and Sauer [62], p. 119). Future research could also explore the type of learning that occurs in dynamic situations and if transfer learning happens in such tasks [63]; and what tools would facilitate learning in a virtual management simulation [64]. Participants could retake the simulation after critical effective feedback is provided [65] or they could work on a task with different characteristics (e.g., the simulation of a platoon commander). Haerem and Rau [66], for example, showed that different task characteristics lead to different task perceptions and different performance among experts and novices. To further investigate the validity of micro-worlds, future studies could assess complex knowledge-rich scenarios and multiple complex systems for specific domains [67,68,69].

An open question still refers to the generalizability of simulation data to real-world behavior in general and CPS in particular. Future research could for example investigate the predictive validity of simulation performance and real-world performance.

## 5. Conclusions

This study contributes to the lack of empirical research on successful and failing CPS strategies in business experts and novices, the influence of prior knowledge and crystallized intelligence on CPS as mentioned in the Introduction to this Special Issue on Intelligence and CPS [70]. Additionally, this study sheds light on the contradicting expert-rigidity versus expert-flexibility models. Although decision-making strategies in business experts and novices have been described in case studies [71] and in the field of cognitive psychology and engineering [72,73,74], as well as in the field of politics [75], they have been rarely investigated empirically and validated by connecting them to performance.

The findings related to expert-novice comparisons are especially interesting, relevant and directly applicable to training programs in CPS. Danner and colleagues [76] suggest that CPS tasks are helpful for choosing prospective employees and for improving decision-making expertise utilized in day-to-day job functioning. A meta-analysis reviewing 65 studies has shown that computer-based simulations are more effective than other instructional methods [77].

Novices could learn from the successful strategies of experts. For example, novices can learn not to engage in action right away, but to, instead, spend time analyzing a problem situation and to always be aware that the situation can change as decisions must be adopted.

The results of the current study together with research on crystallized intelligence and expert-novice differences [20] show that performance differences are related to the degree of business expertise and successful business CPS strategies acquired by the expert over years. Results also show how beneficial it is to observe CPS behavior and changes over time. Contrary to some models claiming rigidity of experts [23], in the current study, the key difference between experts and novices was the ability of experts to adapt and change their decisions according to the changes in the situation, speaking to expert-adaptability models. This is exactly one of the key demands of jobs in the current times [78,79,80], where the professional world is changing at a rapid pace and people have to always be prepared to adjust to changes. A manager reacts, then observes the decision outcome and as a consequence, adjusts the decisions. This can be summarized in the key strategy attributed to Napoleon “On s’engage et puis on voit.” (You commit to something/engage and then you see.) (Baring [81], p. 47; Lenin [82], p. 480).

## Figures and Tables

**Table 1:** Analyses of variance results.

	Wilk’s Lambda	*F*	*df*	*p*	*ƞ*^2^_p_
Advertising expenses					
Group × Month	.60	1.66	(36, 206)	.02	.23
Month	.60	3.83	(18, 103)	<.001	.40
Group	-	.01	(2, 120)	.99	.00
Market research expenses					
Group × Month	.68	1.15	(36, 198)	.27	.17
Month	.61	3.57	(18, 99)	<.001	.39
Group	-	1.63	(2, 116)	.20	.03
Personnel expenses					
Group × Month	.66	1.36	(36, 210)	.09	.19
Month	.78	1.68	(18, 105)	.05	.22
Group	-	1.50	(2, 122)	.23	.02
Advertising changes	-	21.72	(2, 137)	<.001	.24
Market research changes	-	3.92	(2, 137)	.02	.05
Personnel changes	-	16.78	(2, 135)	<.001	.20
Initial problem exploration time (first 2 months)	-	6.33	(2, 137)	.002	.09

**Table 2:** Means and standard deviations of decision-making variables and their correlations with performance also indicating partial correlations.

	Performance	Performance, Controlled for Age and Gender	*M*	*SD*
Performance: Total monies at Month 19	-	-	157,428.63	1,699,632.51
Exploration: Time for first two months	.05	.05	20.55	11.92
Total months completed	−.04	−.04	23.03	3.02
Decision making: Mean of expenses for advertising (1 to 19)	−.17 *	−.17 *	10,777.54	22,260.11
Decision making: Mean of expenses for market research (1 to 19)	−.04	−.004	1,593.22	1638.49
Decision making: Mean of expenses for personnel (1 to 19)	−.28 **	−.30 **	113,179.74	32,328.23
Advertising changes	.17 *	.15	.43	.27
Market research changes	−.15	−.12	.34	.20
Personnel changes	.37 ***	.35 ***	.73	.19

Note: The values of the last three variables indicating changes are relative frequencies always divided by the total number of CHOCO-FINE months played. *** *p* < .001; ** *p* < .02; * *p* < .065.

**Table 3:** Regression analyses predicting performance in CHOCO-FINE.

	Standardized Beta	*t*	*p*	95% Confidence Interval for B	
				Lower	Upper
Age	−0.07	−0.75	.46	−38,536.21	17,397.34
Gender	0.21	2.46	.02	140,710.90	1,303,929.19
Exploration: Time for first two months	0.11	1.25	.22	−10,275.91	45,293.20
Total months completed	−0.02	−0.25	.81	−398,608.24	310,715.24
Total advertising expenses	−0.11	−1.30	.20	−20.71	4.31
Total market research expenses	0.07	0.61	.54	−161.15	304.72
Total personnel expenses	−0.28	−3.14	.002	−23.21	−5.25
Advertising changes	0.13	1.48	.14	−275,343.05	1,882,048.28
Market research changes	−0.04	−0.30	.77	−2,221,426.93	1,642,531.64
Personnel changes	0.36	4.01	<.001	1,584,115.59	4,675,047.85
